# A pilot study investigating the safety and feasibility of endoscopic dilation using a radial incision and cutting technique for benign strictures of the small intestine: a study protocol

**DOI:** 10.1186/s40814-022-01046-8

**Published:** 2022-04-19

**Authors:** Rintaro Moroi, Hisashi Shiga, Kotaro Nochioka, Yusuke Shimoyama, Masatake Kuroha, Yoichi Kakuta, Yoshitaka Kinouchi, Atsushi Masamune

**Affiliations:** 1 Division of Gastroenterology, Tohoku University Hospital, 1-1, Seiryo, Aoba, Sendai, Miyagi 980-8574 Japan; 2 Clinical Research, Innovation and Education Center, Tohoku University Hospital, Sendai, Miyagi Japan

**Keywords:** Radial incision and cutting, Endoscopic dilation, Small bowel stenosis

## Abstract

**Background:**

Small benign intestinal stenosis is usually treated by endoscopic balloon dilation (EBD) or surgery. Although EBD and surgery are able to resolve the stenosis in most cases, they are associated with several problems such as insufficient dilation and surgical stress, respectively. On the contrary, a novel approach called radial incision and cutting (RIC) is reported to have several benefits when compared to EBD and surgery. We can currently adopt RIC only for the strictures in the colon or terminal ileum and not for those stenotic lesions present further in the small intestine where balloon-assisted endoscopy is utilized, because the long-type electric knife is currently not approved for use in Japan. We will herein conduct a pilot study to investigate the safety and feasibility of RIC for treating the benign stenoses of the small intestine using the long-type electric knife.

**Methods:**

This will be a single-center, single-arm, interventional trial. The major criteria for inclusion will be age ranging from 20 to 80 years and the presence of benign stenosis in the small intestine. We will perform RIC on 10 participants. The primary outcome is the safety of this procedure, which will be assessed by measuring the frequency of adverse events of special interest. The secondary outcomes will be technical success rate, improvement in subjective symptoms, procedure time, and duration of hospitalization.

**Discussion:**

This pilot study will provide useful information that will aid in adopting RIC for treating the benign strictures present in the small intestine.

**Trial registration:**

jRCT Identifier, jRCTs022200040. Registered on 1 March 2021.

**Supplementary Information:**

The online version contains supplementary material available at 10.1186/s40814-022-01046-8.

## Background

Benign stenosis of the lower gastrointestinal tract is a common complication that physicians often encounter in clinical practice. These stenotic lesions develop due to various causes such as stricture formation at the anastomotic site after lower gastrointestinal tract surgery due to colorectal cancer [1], inflammatory bowel disease (IBD) [2, 3], and diverticulitis [4]. Other diseases and conditions, including mucosal healing in IBD, ischemic colitis [5], enteritis caused by drugs [6], and endoscopic mucosal resection of a large colorectal tumor [7], can also cause such stenotic lesions. The standard therapies for benign stenosis of the lower gastrointestinal tract are endoscopic balloon dilation (EBD) [8–11] and surgery, including resection of the stenosis [12] or strictureplasty [13]. Although surgery can definitely resolve the stenosis compared to another therapy, it is accompanied by a significant surgical stress. Some patients, especially those suffering from Crohn’s disease, develop re-stenosis and require multiple surgeries, which leads to short bowel syndrome [12, 13]. Thus, surgery should be avoided, if possible. On the contrary, EBD shows good results with a higher technical success rate and improvement in symptoms [8–11]. However, about half of the patients who undergo EBD develop re-stenosis after 3 years [9]. EBD also needs to be performed several times to obtain a certain dilation diameter, which prolongs the duration of the hospital stay. Furthermore, the degree of dilation achieved after EBD is relatively small. A prospective study reported that the rate of scope passage after EBD was only 76.3%, despite the technical success rate of 93.7% [10]. Therefore, other novel approaches for treating the benign stenosis of the lower gastrointestinal tract are required.

We previously reported the efficacy of the radial incision and cutting (RIC) method for intestinal strictures in five patients with Crohn’s disease (CD) as a pilot study [14]. RIC was initially reported as a dilation technique for refractory stenoses that developed after surgical resection of esophagogastric diseases. In this technique, the stricture is incised with an endoscopic electrical knife [15]. We adopted this method for benign stenoses of the lower gastrointestinal tract, namely those developing at the anastomotic site after ileocecal resection and rectal stenosis due to mucosal healing. Our pilot study showed a higher technical success rate (100%) and a shorter duration of hospitalization compared to EBD.

We have been conducting a continuous RIC study, which is scheduled to recruit 20 patients with benign stenosis in the lower gastrointestinal tract (UMIN000039411). We have achieved technical success in 17 cases (unpublished data). Although, delayed bleeding occurred in several cases, we secured hemostasis endoscopically in all the cases without requiring blood transfusion. RIC was also able to achieve larger diameters after dilation compared to EBD. As previously mentioned, RIC has several merits when it is used to treat benign stenosis in the lower gastrointestinal tract. However, the regions of the gastrointestinal tract for which we can adopt RIC are restricted to the colon, rectum, and terminal ileum because of the short working length of the endoscopic electric knife approved for use in Japan. The length of the endoscopic electric knife, which we can obtain in Japan, is 1850 mm, and this length is too short to reach further into the small bowel with balloon-assisted endoscopy (BAE) (working length 2000 mm). Therefore, we cannot conduct RIC for the distant stenotic lesions of the small bowel. On the contrary, a long-type electric knife with a working length of 2300 mm (FlushKnife BTS long-type, FUJIFILM, Tokyo, Japan) is approved for use in the USA, but not in Japan. If we use BAE with the long-type electric knife, we would be able to utilize RIC for all segments of the lower gastrointestinal tract. If the indication of use of RIC is expanded to include the patients with small bowel stenosis, surgery can be avoided. We herein plan to conduct a pilot study for using RIC in the treatment of small bowel stenoses.

This study aims to investigate the safety and feasibility of utilizing RIC for benign stenoses of the small intestine using the long-type electric knife, which is not approved for use in Japan.

## Methods

### Study design

**Fig. 1:**
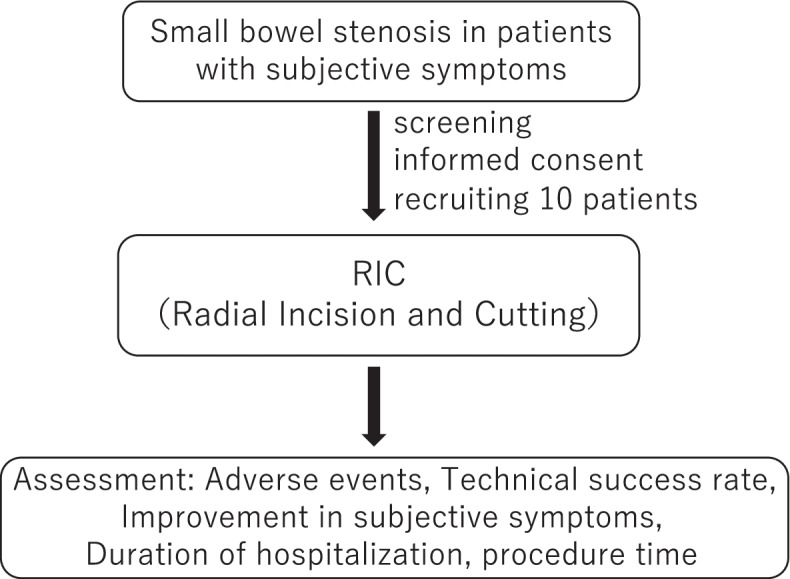
Flowchart describing the study protocol. This study will target small bowel stenosis in patients with subjective symptoms. RIC will be performed in ten participants. The primary outcome is the safety of RIC being evaluated by the frequency of adverse events

This study will be a pilot study for a prospective single-center, single-arm, interventional trial. The flowchart of the study is shown in Fig. 1.

### Approvals

The trial has been approved by the institutional review board of the Tohoku Certified Review Board of Tohoku University (Ministry of Health, Labor and Welfare Certified Clinical Research Review Board, Tohoku University) and is registered at jRCT (Japan Registry of Clinical Trial; jRCTs022200040, URL: https://jrct.niph.go.jp/re/reports/detail/11533).

### Setting

**Fig. 2:**
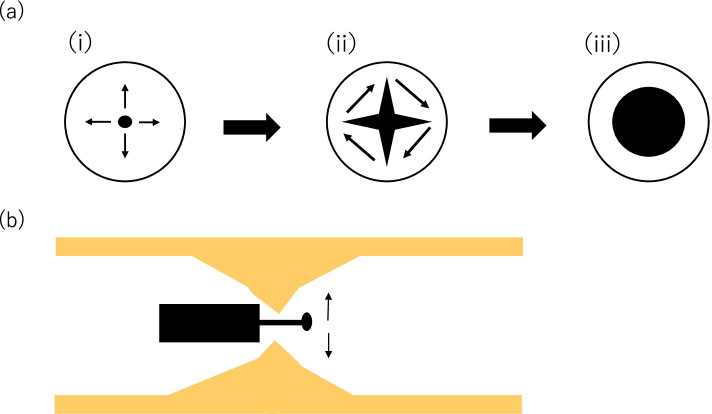
A The scheme of RIC from front view. Radial incision (i). Horizontal cut (ii). Complete dilation (iii). B The scheme of RIC from side view

This study will be conducted at a single center (Tohoku University Hospital) where the investigators are well experienced in the RIC technique. Tohoku University Hospital is a tertiary referral center which serves high-level medical treatment for mainly Miyagi prefecture covering 2,000,000 people. Our division has about 2000 patients with inflammatory bowel disease and conducting endoscopic submucosal dissection (ESD) for colorectal tumors. RIC technique resembles ESD in terms of using an electric knife. The scheme of RIC is shown in Fig. 2. To our knowledge, only two institutes conduct a trial of RIC in Japan. The eligible operator is an endoscopist who experienced ESD in more than 50 cases.

### Population

We will include 10 patients with small bowel stenosis. This is a pilot study to investigate the safety and feasibility of RIC for small bowel stenosis; thus, the number of patients should be as small as possible. However, a certain number of participants are necessary to evaluate the safety and feasibility. Therefore, we have set the number of patients that comprise the study population at 10.

### Inclusion criteria

All the participants should meet the inclusion criteria as follows:

1. Patients of any sex aged between 20 and 80 years
2. Patients with benign small bowel stenosis
3. Patients with performance status between 0 and 2
4. Patients who are able to provide written informed consent for participating in the study

### Exclusion criteria

Participants with any of the following conditions will be excluded:

1. Length of stenosis greater than 2 cm
2. Presence of abscess or fistula close to the stenosis
3. Presence of edematous rather than fibrotic stenosis
4. Malignant stenosis
5. Taking any anti-coagulant or anti-platelet drugs that cannot be discontinued temporally
6. Pregnancy (including those in whom pregnancy is suspected), within 28 days after childbirth, or actively lactating
7. History of psychiatric disease
8. History of severe cardiopulmonary disease
9. Those who are deemed ill-suited to the study by the lead doctor

### Trial intervention

We will perform the RIC procedure in a way that is similar to that reported in previous studies [14–20]. The endoscopic electric knife used in this trial will be a Flushknife BTS long-type (FUJIFILM Corporation, Tokyo, Japan), the use of which is not covered by insurance in Japan. Although we have not specified a particular BAE for this trial, we usually use a double-balloon endoscope (FUJIFILM Corporation, Tokyo, Japan), or a single-balloon endoscope (Olympus Medical Science, Tokyo, Japan) in Japan. The schedule of RIC is shown in Table 1.

**Table 1:** Schedule of the pilot study out-hospital

	Out-hospital		Out in-hospital	In-hospital					Out-hospital	
	Obtain consent	Screening-1	Screening-2	Enrollment	Ric	Day 1	Day 2	Day 7	Day 28	Discontinuation
Allowance duration (day)	−90 ~ −2 days	−90 ~ −2 days	−35 ~ −2 days	−2 ~ 0	±2 ~ 02	±2 ~ 02	±2 ~ 02	±2 ~ 02	±2 ~ 02	±2 ~ 02
Obtain consent	●									
Enrollment				●						
Ric					●					
Patients’ background			●							
Balloon-assisted small bowel endoscopy			●							
Computed tomography		●^d^								
Electro-cardiogram		●^d^								
Colonoscopy		▲^d^								
Abdominal ultrasound		▲^ed^								
Magnetic resonance imaging		▲^ed^								
Abdominal X-ray						●				
Body examination			●		●	●	●	●	●	●
Peripheral blood examination^a^			●			●	●		●	●
Biochemical examination^b^			●			●	●		●	●
Prgnancy test^c^			●							
Concomitant medication			●		●	●	●	●	●	●
Questionnaire: VAS			●						●	
Questionnaire: CDAI			●						●	
Adverse events					●	●	●	●	●	●

*RIC* Radial incision and cutting, *VAS* Visual analog scale, *CDAI* Crohn’s disease activity index

^a^Peripheral blood test: white blood cell, red blood cell, hemoglobin, hematocrit, platelet cell, erythrocyte sedimentation rate

^b^Biochemical test: total bilirubin, aspartate transaminase, alanine aminotransferase, alkaline phosphatase, gamma-glutamyltransferase, serum iron, total protein, albumin, total cholesterol, c-reactive protein

^c^For women with age of fertility

^d^Whether own or another facility is allowed

^e^Select either one

### Outcomes

#### Primary outcome

The primary outcome of this study is the safety of using RIC for benign stenosis in the small intestine, and the safety is evaluated by measuring the frequency of adverse events of special interest (AESI) during 1 month after RIC. AESI includes complications related to RIC, such as delayed bleeding and perforation, which require endoscopic hemostasis or surgery. Safety of RIC is selected as the primary outcome because this is a pilot study and there are no other studies reporting the safety of performing RIC in the small intestine using BAE.

#### Secondary outcomes

- The technical success rate of RIC (defined by the rate of scope passage at the dilation site just after completion of RIC)
- Improvement in subjective symptoms, which will be evaluated using the visual analog scale (VAS) or the Crohn’s disease activity index between before and after RIC
- Duration of hospitalization
- Procedure time of RIC which is from the start to the end (completion of dilation) of RIC

We have selected these secondary outcomes to evaluate the feasibility and efficacy of performing RIC in the small intestine in this trial.

### Data collection

All data will be entered electronically using a case report form which includes age, sex, an underlying disease which contributed the to the formation of stenosis, medication, past history, smoking history, examinations to evaluate eligibility (including laboratory data, radioscopy, endoscopy, computed tomography, magnetic resonance imaging, and abdominal ultrasound), pictures, and movies of RIC. That information is considered reliable and valid to evaluate the feasibility and safety of RIC for the small intestine.

### Data management and statistics

As with data collection, standard processes will be implemented to improve the accuracy of data entry and coding. A formal hypothesis testing is not appropriate because this is a pilot study to investigate the feasibility and safety of RIC for the small intestine with a small sample size. Descriptive statistics for primary and secondary outcomes described above will be conducted. We will evaluate the improvement of subjective symptoms between before and after RIC using Student’s *t* test and presenting 95% confidence intervals.

### The study procedure and schedules

The SPIRIT checklist was followed as a guide for reporting this study protocol. It is shown in Table 1. The populated SPIRIT checklist is also provided as an additional file (see Additional file 1). We will target and screen those patients who present with subjective symptoms such as abdominal bloating, vomiting, and ileus. Most of the screening examinations will be clinical. Abdominal ultrasound or magnetic resonance imaging will be employed to investigate the wall thickness at the stenotic site as much as possible. The investigators will explain the procedure of the trial to those patients who meet the criteria and will obtain written informed consent. Participants will be able to withdraw their consent to participate whenever they want. Patients’ registration will be performed using the eligibility confirmation sheet. This pilot study is a single-arm non-randomized trial; therefore, the enrollment will be accomplished after filling out the confirmation sheet.

The enrolled participants will undergo the RIC procedure and will stay admitted to the hospital for around 7 days after RIC. Follow-up will be conducted after 1 month. The occurrence of any adverse events (AEs) will be monitored during this time. Subjective symptoms including abdominal bloating, abdominal pain, and nausea will be evaluated using VAS.

### Randomization and blinding

Not applicable.

### Safety

As mentioned above, the safety of RIC is the primary outcome of this study. Bleeding and perforation after RIC are the probable major AEs. Our previous report and other studies showed that delayed bleeding and perforation were the major AEs. The participants could also develop pancreatitis after EBD via the transoral approach. These AE and other complications will be monitored until 1 month after RIC and recorded in a case report form.

Serious adverse events (SAEs) are defined as follows:

1. All deaths and immediate life-threatening events, whether related or unrelated to the procedure
2. Prolongation of hospitalization
3. Substantial disruption of normal life functions

All SAEs will be recorded as well and reported to the institutional review board and the Ministry of Health, Labor and Welfare as soon as possible.

### Monitoring

The principal investigator will designate a monitor to survey if the investigators obey the study protocol. The monitor will also survey the safety and adverse events of study participants.

## Discussion

This paper describes the protocol for a pilot study that explores the safety and feasibility of RIC for small bowel stenosis. To the best of our knowledge, there are no reports in which RIC is performed for benign stenosis in the small intestine using BAE. Our previous study [14], the ongoing trial, and other studies target those lesions that are located in the colon and terminal ileum where a colonoscope can reach easily. The RIC technique, according to the previous studies [14, 16, 19, 20], could provide several benefits such as larger dilation and a shorter hospital stay when compared to EBD. However, we are unable to obtain the long-type electric knife, which has enough length so that RIC can be performed in the small intestine using BAE. If we are able to adapt this RIC technique for small intestinal stenosis for which BAE is required, surgery could be avoided in these patients. We have performed the RIC technique in over twenty patients, and it was successful in all the cases. Therefore, we may be able to perform this technique without encountering significant technical challenges.

On the contrary, RIC showed a relatively high frequency of delayed bleeding compared to EBD (8.8 vs. 0%) [19]. This important issue should be addressed in the future. Therefore, we selected the safety of RIC as a primary outcome in this pilot study. We can evaluate the safety by investigating AESI during the 1-month follow-up after RIC. We hope that this pilot study would be useful in establishing the safety of RIC for small bowel stenosis. If we are able to confirm that performing RIC in the small intestine is relatively safe, we will conduct a continuous study with a larger number of patients.

### Limitation

The number of patients in this trial is small because this is a pilot study.

### Trial status

This trial study has been approved. We plan to start recruitment in January 2021 and end it in June 2024.

## Supplementary Information

**Additional file 1.** Populated SPIRIT Checklist. Detailed checklist describing the proposed sequence of events.

## Data Availability

Data available on request from the authors.

## Abbreviations

AE: Adverse events
AESI: Adverse events of special interest
BAE: Balloon-assisted endoscopy
EBD: Endoscopic balloon dilation
ESD: Endoscopic submucosal dissection
RIC: Radial incision and cutting
SAE: Serious adverse event
VAS: Visual analog scale
